# VennPlex–A Novel Venn Diagram Program for Comparing and Visualizing Datasets with Differentially Regulated Datapoints

**DOI:** 10.1371/journal.pone.0053388

**Published:** 2013-01-07

**Authors:** Huan Cai, Hongyu Chen, Tie Yi, Caitlin M. Daimon, John P. Boyle, Chris Peers, Stuart Maudsley, Bronwen Martin

**Affiliations:** 1 Metabolism Unit, National Institute on Aging, National Institutes of Health, Baltimore, Maryland, United States of America; 2 Receptor Pharmacology Unit, National Institute on Aging, National Institutes of Health, Baltimore, Maryland, United States of America; 3 Institute for Cardiovascular Research, Leeds Institute of Genetics, Health and Therapeutics, University of Leeds, Leeds, West Yorkshire, United Kingdom; University College Dublin, Ireland

## Abstract

With the development of increasingly large and complex genomic and proteomic data sets, an enhancement in the complexity of available Venn diagram analytical programs is becoming increasingly important. Current freely available Venn diagram programs often fail to represent extra complexity among datasets, such as regulation pattern differences between different groups. Here we describe the development of VennPlex, a program that illustrates the often diverse numerical interactions among multiple, high-complexity datasets, using up to four data sets. VennPlex includes versatile output features, where grouped data points in specific regions can be easily exported into a spreadsheet. This program is able to facilitate the analysis of two to four gene sets and their corresponding expression values in a user-friendly manner. To demonstrate its unique experimental utility we applied VennPlex to a complex paradigm, *i.e.* a comparison of the effect of multiple oxygen tension environments (1–20% ambient oxygen) upon gene transcription of primary rat astrocytes. VennPlex accurately dissects complex data sets reliably into easily identifiable groups for straightforward analysis and data output. This program, which is an improvement over currently available Venn diagram programs, is able to rapidly extract important datasets that represent the variety of expression patterns available within the data sets, showing potential applications in fields like genomics, proteomics, and bioinformatics.

## Introduction

The present biomedical dominance of mass analytical “*omics*” fields, such as genomics, proteomics, and metabolomics, have resulted in the facile creation of highly complex data sets with often unwieldy numbers of data points [1], [2]. Venn analysis often forms the first filtration step for complex and interconnected data corpora. Therefore simple mechanisms for the coherent separation of mass data sets, *e.g.* microarray mRNA transcript expression profiling studies, in which a large amount of genes may possess distinct regulation polarities, greatly assist data set interpretation [2], [3]. Analyzing these gene datasets requires the appreciation not only of gene identity itself, but also the polarity of expression regulation for each transcript (*i.e*. the changing pattern of each gene). Venn diagrams are the most commonly used method to compare and visualize datasets of diverse groups [4]–[6]. Venn diagrams typically consist of intersecting circles or ellipses, which represent the different data set groups, and the overlap and separation between the circles allows for a visual representation of the logical relationships between different data sets [7]–[9]. Despite the scientific popularity of Venn diagram-mediated data separation, alternative methodologies do exist. For example, Euler diagrams, first introduced by Leonhard Euler, can be used to illustrate set-theoretic relationships, *i.e.* intersection, subset and disjointedness. Venn diagrams themselves can be considered more restrictive forms of Euler diagrams as all sets in the Venn diagrams must intersect. Furthermore, Venn diagrams must contain all 2*^n^* logically possible zones of overlap between its *n* closed curves representing *n* sets, while some zones might be missing if they are empty in Euler diagrams. However for the majority of biomedical scientists standard Venn diagrams have been the primary mechanism by which complex interconnected datasets can be analyzed.

Currently, various Venn diagram programs are freely available to assist in the facile visual interpretation of biological datasets. Widely employed examples of such free-to-use programs include Pangloss Venn diagram generator (http://www.pangloss.com/seidel/Protocols/venn4.cgi), Venn Diagram Plotter (http://omics.pnl.gov/software/VennDiagramPlotter.php) and Venny (http://bioinfogp.cnb.csic.es/tools/venny/index.html) [10]. BioVenn is also a web application for comparison and visualization of biological lists using area-proportional Venn diagrams (http://www.cmbi.ru.nl/cdd/biovenn/) [7]. Other programs such as GeneVenn (http://genevenn.sourceforge.net/) [9] and VennMaster (http://www.informatik.uni-ulm.de/ni/staff/HKestler/vennhyp/) [5], [8] possess the additional feature of linking genes within each group to related information in the NCBI Entrez Nucleotide database or the Gene Ontology database (http://www.geneontology.org/) [11]. Interestingly, VennMaster is also able to generate area-proportional Euler diagrams of its output. In general, there is no perfect solution for these area-proportional Euler diagrams using circles or regular polygons, however using VennMaster optimally proportional representations can be generated using swarm and evolutionary optimization algorithms [5]. The application ‘VennDiagram’ (http://www.biomedcentral.com/1471-2105/12/35) [4] enables the automated generation of highly-customizable, high quality Venn and Euler diagrams in the R statistical environment. In addition to these platforms, our recently developed high-content Venn diagram program, VENNTURE (http://www.grc.nia.nih.gov/), is able to visualize up to 6 datasets at one time and also facilitate multiple forms of readily-usable output [12]. While many of these exemplary and well-written programs allow the facile Venn-based segregation of multiple datasets, limitations however of these packages do exist, *e.g*. a lack of a capacity for the representation of extra complexity between datasets that contain denominators with diverse numerator values, such as the regulation pattern difference for gene expression in each group. Considering that variation in numerator polarity between diverse test groups is one of the most widely applied analyses employed for microarray-generated gene datasets, this is surprising. Without this feature further effort to understand and illustrate the complex and subtle differences between the data sets may be required. In this paper, we describe the development of a simple Venn diagram-generating program, VennPlex. VennPlex possesses important features such as: user-friendly data importation and exportation; compatibility with commonly-used windows-based programs, and most importantly, and highlighting the novelty of VennPlex, the capacity to facilitate the analysis of two to four sets of any length with the ability to report upon diverse numerical regulation polarities for multiple shared factors between sets. Compared to our previous diagram-generating program VENNTURE, VennPlex is a unique piece of software addressing a different mathematical problem. VENNTURE is a traditional Edwards Venn diagram-generating program that is capable of comparing and visualizing up to six sets. VennPlex can analyze data contained in two to four data sets where the factors being separated may possess various numerator values of distinct polarities. Here we demonstrate the performance of VennPlex using data sets representing the transcriptomic responsiveness of primary cortical astrocytes to various ambient cultured O_2_ tensions. While considerable attention in the central nervous system (CNS) is paid to neuronal activity, glial cells in the CNS possess equally important functional roles in regulating brain metabolism and electrical activity. Astrocytes are a specialized form of CNS glial cells and are of major importance in the control of cerebral blood flow and, hence, brain O_2_ levels [13], [14]. With respect to transcriptional activity, ambient O_2_ levels have been recognized as a major determinant of gene expression in all tissues examined to date, including the CNS [15], [16]. Protracted periods of poor blood flow, inducing functional hypoxia, has been shown to induce profound CNS cellular damage and increase the likelihood of developing progressive dementias such as Alzheimer’s disease (AD) [17]–[19]. Therefore, systematic and unbiased studies of the responsiveness of CNS astrocytes to various ambient O_2_ tensions, at the transcriptomic level, may assist in the development of anti-neurodegenerative therapeutic strategies [20]. We have demonstrated previously that variant O_2_ tensions induce specific and physiologically-focused transcript regulation signatures in primary cortical astrocytes [20]. However, using existing Venn diagram analyses of oxygen tension-dependent transcriptional responses in astrocytes only indicated the total number of transcripts which were altered uniquely or commonly between the different experimental oxygen tension groups (1, 4, 9% ambient O_2_). Existing, free-to-use programs were unable to assist in the visualization of transcriptional regulatory patterns of the genes controlled by different oxygen conditions. VennPlex, with its novel feature of being able to visualize contra-regulated datapoints, facilitates a more comprehensive and interconnected appreciation of the O_2_-related genomic response patterns present in cortical astrocytes. We demonstrate here that VennPlex is able to: import and export data easily, (especially the exportation of numerical data within each region in the Venn diagram); draw multiple circle sets and present the transcriptional regulation patterns such as commonly or contra-regulated genes in each intersection in a readily-usable format.

## Materials and Methods

### VennPlex Software

VennPlex version 1.0.0.2 was created using Microsoft Visual C#. VennPlex can be used on any personal computer using Microsoft Windows™ XP or higher operating system. For users without access to MicroSoft-Office™-based platforms VennPlex can also be employed using the OpenOffice platform (http://www.openoffice.org/). VennPlex can generate from two to four set Venn diagrams using denominators with discriminative numerator values. VennPlex allows the ability to identify individual factors that are up-, down- or contra-regulated, with respect to their associated numerators, between two or more datasets.

### VennPlex Availability

VennPlex version 1.0.0.2 can be accessed freely at the dedicated National Institute on Aging URL of http://www.irp.nia.nih.gov/bioinformatics/vennplex.html. This software is available for download and is contained in a Winzip file along with the NIH Software Transfer Agreement, a test sample dataset and a PDF formatted copy of the journal article. For any further information regarding the transfer of material pertaining to VennPlex we encourage researchers to enquire at the following website, http://ttc.nci.nih.gov/forms/mta.php.

### VennPlex Data Processing

**Figure 1:**
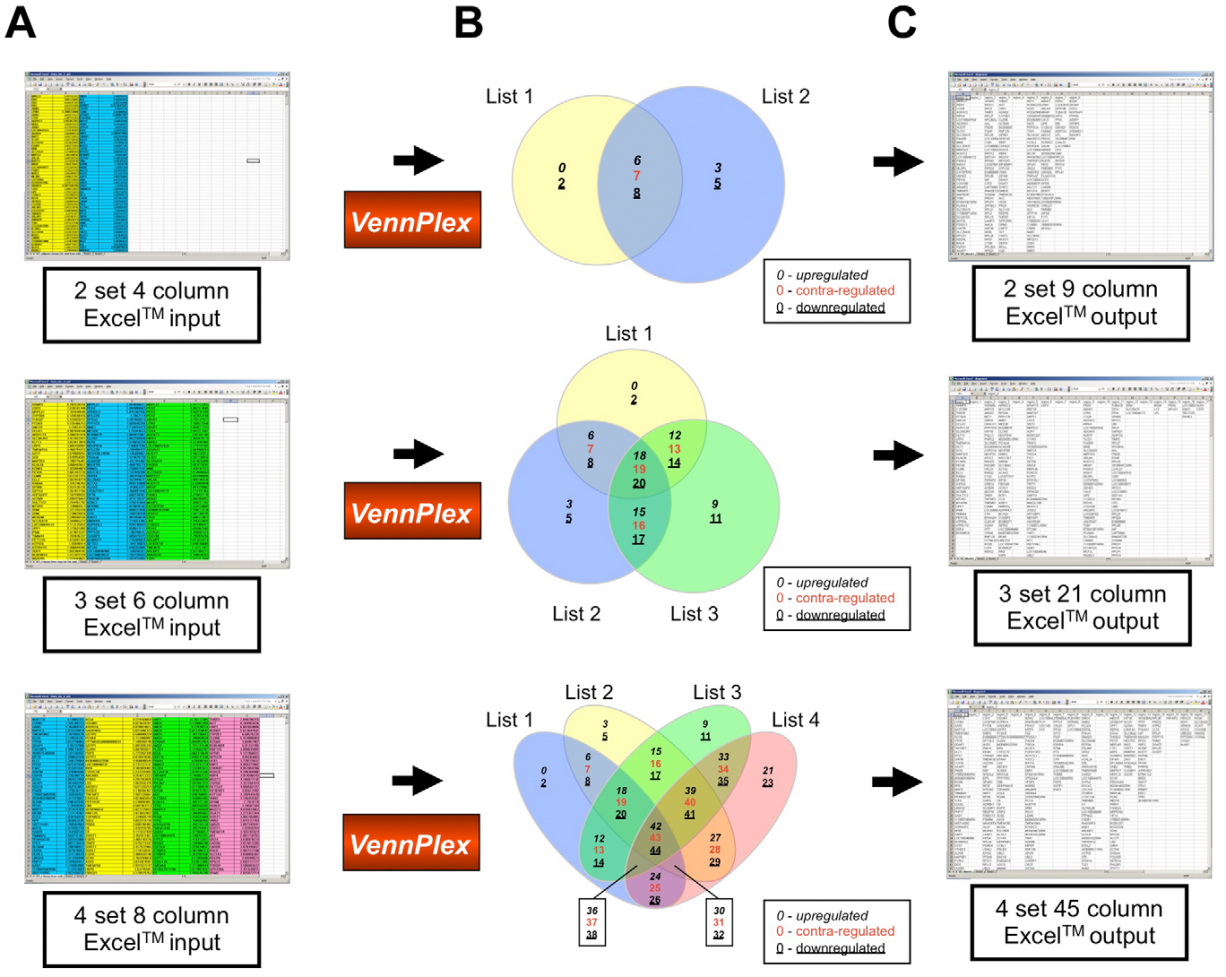
VennPlex data input and output scenarios. (A) Data input in Excel™ files. Two gene sets and corresponding z-ratios were organized into an Excel™ input file and highlighted in yellow and blue color separately. Three set input data were highlighted in yellow, blue, and green accordingly. Four set input data were highlighted in yellow, blue, green, and red, respectively. (B) Venn diagram drawn by VennPlex program. The numbers in the different sections of 2, 3, 4-set Venn diagram indicate the output set orders in the resultant extracted datasheet in panel (C). To indicate the polarity of numerical regulation of each factor within each set or intersection we have employed a two or three-way key system. The number of factors unique to a set, with a positive regulation polarity are identified with an *italic* numeral. The number of factors unique to a set, with a negative regulation polarity are identified with an underlined numeral. The number of factors common between multiple sets are indicated with a red colored numeral. This is indicated in a box key for each panel in (B). (C) Data output in Excel™ files.

VennPlex assigns a bit in a 32-bit integer to each input data set (2–4 sets) that VennPlex detects. Possible values of the 32-bit integer correspond to all possible regions in the resulting diagram. VennPlex then converts the datasets into a hashset of unique strings and then completes a single pass through the hashset, incrementing all associated bits in the 32-bit integer. The region selected by the 32-bit integer is then incremented, and at this point, the differentiation between up-regulation, down-regulation, and ‘*contra-regulation*’ (diverse regulation polarity values for one specific text between different input sets) is made based upon the z-ratios. VennPlex then draws the appropriate Venn diagram contained in the Excel™ file (Figure 1). This algorithm takes O(N * S) time, where N is the number of elements in the longest column and S is the total number of Venn diagram groups. If two or three data sets are uploaded, proportional circular sets will be drawn; for four sets, non-proportional ellipses will be used to preserve diagram symmetry. Diagrams for four sets use a premade template. Generation of sets for each region of the Venn diagram follow the naïve implementation of the generic Inclusion-Exclusion Principle, as follows:
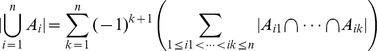



The algorithm for computing this for n sets is O(n!) time, but is effectively constant for small sets. The principle is used regardless of the number or cardinality of sets. For three or fewer sets, we compute the radii of each individual circle in the Venn diagram such that the areas of the circles are proportional to their respective cardinalities. The centers of each circle are then iteratively moved until there is sufficient space in the overlap between two regions to print text. For four sets, a premade template is used, on top of which text is displayed. The text for all sets is determined by the aforementioned algorithm.

### VennPlex Data Input and Output Results

To upload any form of biological data, with a textual identifier (*e.g*. Official Gene Symbol) and associated numerical factor (*e.g.* z-ratio) into VennPlex, the data sets must be in either Microsoft Excel™ Compatibility Mode (*.xls) or Comma Separated Value (*.csv) format. All Excel™ files require that each column of gene name or gene accession number be followed by a column of corresponding z-ratios. The first two columns containing gene set and related z-ratio values in any given spreadsheet represents the first set and all subsequent pairs of columns represent each additional set. All CSV files must be comma-delimited and all cells must be arranged in the exact manner as that of Excel™ files. To upload data, select “Load from.csv” or “Load from.xls” from the File menu. After the appropriate file is loaded, VennPlex then automatically detects how many gene sets are present in the input file. If simple geneset data is employed (Gene Symbols with associated z-ratio values) then the Venn diagram will be instantly populated with the input gene-regulation data. The *italic* numbers of numerical categories represent the number of up-regulated genes; the underlined numbers represent the down-regulated genes; the red numbers represent the ‘contra-regulated’ genes (Figure 1). The data can either be exported as a CSV or Microsoft Excel™ Compatibility Mode file using the “Save as.csv” and “Save as.xls” buttons on the File Menu. In the resulting file, the first row indicates all possible regions on the Venn diagram. These regions are uniquely identified by a positive integer. To view the region identification numbers, click the “Sequence” button. To switch back to the normal view, click the “Result” button. Each region can then be exported to a Microsoft Excel™ Compatibility Mode file by clicking “Save Region” and entering the specific region number the users are interested in. The exported file contains each gene in the set, along with the z-ratios associated with that gene in each of the regions. The resulting Venn diagram can be exported into a JPEG, Bitmap, GIF, or PNG image using the ‘Save picture’ option in the File menu. Finally, to exit the program, click on “Exit” under the File menu.

### Biological Data Generation

Primary cultures of rat cortical astrocytes were obtained as described previously [21]. Cortical astrocytes were then subjected to 1, 4, or 9% O_2_ tension and also normal cell culture oxygen conditions (20% O_2_) [20]. RNA was then extracted from the cells using the RNEasy Mini Kit (QIAGEN, Valencia, CA) according to the manufacturer’s instructions. The RNA was then examined for quantity and quality using an Agilent Bioanalyzer 2100 (Agilent Technology, Palo Alto, CA). cDNA probe preparation and microarray hybridization were performed as described previously [1], [3]. The microarrays were exposed to phosphorimager screens for 3 days. The screens were then scanned in a Molecular Dynamics STORM PhosphorImager (Molecular Dynamics, Sunnyvale, CA) at 50 µm resolution. Quantification of scanned screens was performed with ArrayPro software. Raw hybridization intensity data were log-transformed and normalized to yield z-scores, which in turn were used to calculate a z-ratio value for each gene with respect to the control tissues. The z-ratio was calculated as the difference between the observed gene z-scores for the experimental and the control comparisons, divided by the standard deviation associated with the distribution of these differences. Z-ratio values ≥ +2.0 or ≤ −2.0 were chosen as cut-off values, defining increased and decreased expression, respectively [1].

## Results

**Figure 2:**
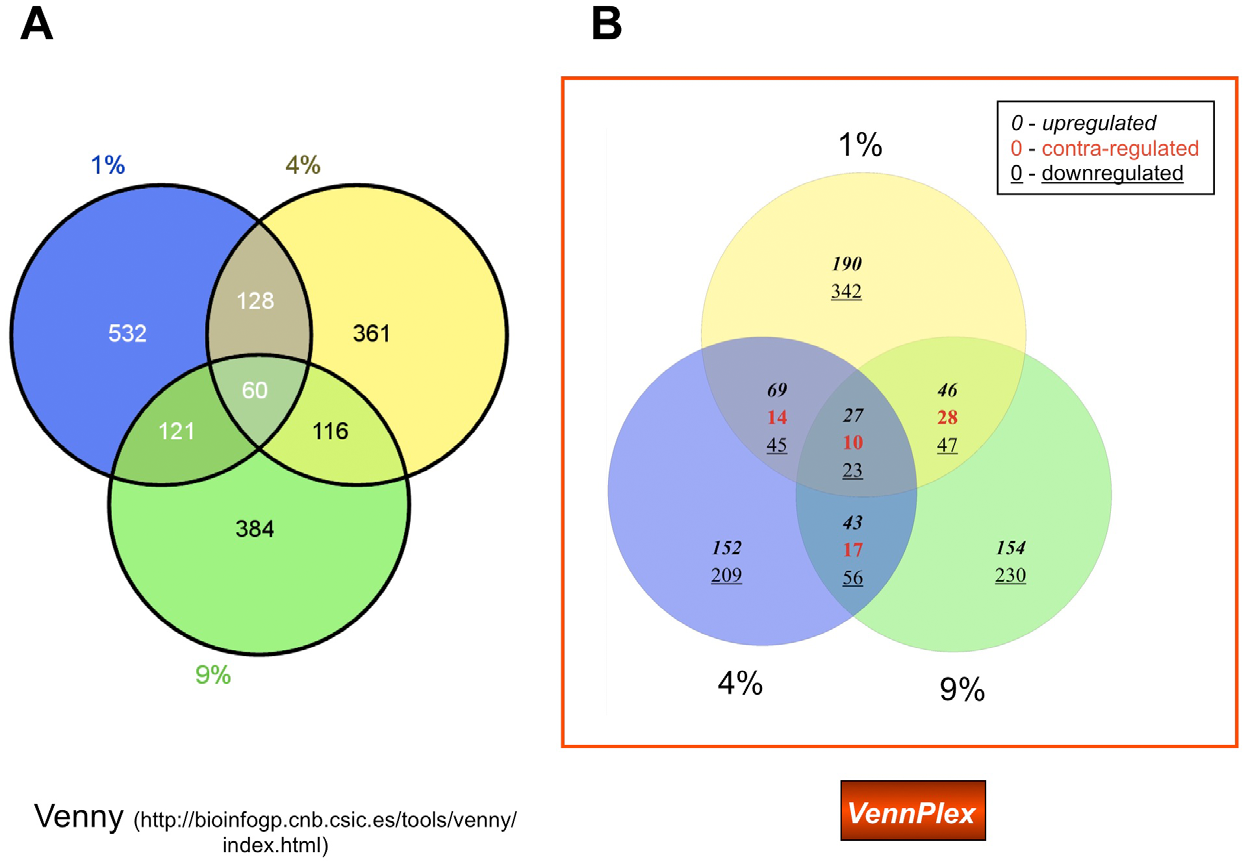
Three-set Venn diagram analysis of multiple oxygen tension-response transcriptional alterations in astrocytes. (A) 3-set Venn diagram drawn by Venny. 1%, 4%, 9% represent 1%, 4%, 9% O_2_ conditions, compared to the 20% O_2_ condition. (B) 3-set Venn diagram generated by VennPlex. The numbers in each section show the number and type (up-, down- or contra-regulated) transcriptional alterations. As stated in Figure 1 the boxed key helps indicate the number of factors in each set or intersection that are upregulated (*italic*), downregulated (underlined) or contra-regulated (red). For example, there are 60 transcripts common to all three sets, 27 are upregulated (*italic*) in all three sets, 23 are downregulated (underlined) in all three sets, and 10 (red) demonstrate a mixture of regulation polarities (contra-regulated) between two of the input sets.

A visual scenario of how VennPlex analyses two, three and four datasets is shown in Figure 1. After different gene sets with corresponding z-ratio values in Excel™ files (Figure 1A) were input into VennPlex, two or three intersecting proportional circles or four set ellipses with numbers of grouped data lists were generated (Figure 1B). It should be noted that the circle sizes, but not the intersections, for up to three sets are proportionally-scaled for the size of the set. All the grouped data lists can be output in one Excel™ file (Figure 1C). Also the gene lists and corresponding z-ratio values in each region (identified as regions from 0 to 44 depending upon the required number of set comparisons: Figure 1B) can be saved in different Excel™ files separately. In order to demonstrate the most important feature of VennPlex, we employed one of our previous gene array data sets of transcriptional response of astrocytes to divergent ambient O_2_ tensions. Significantly (p<0.05) regulated genes were obtained previously by comparing rat primary astrocytes exposed to 1, 4, 9% O_2_ ambient tension to 20% O_2_ tension [20]. A 3-set Venn diagram was drawn in Figure 2A using one of the most commonly-used free-to-use Venn diagram programs, Venny [10], showing the number of genes altered uniquely or commonly in astrocytes exposed to 1, 4, 9% ambient O_2_ tension compared to 20% O_2_ tension. A more sophisticated 3-set Venn diagram was generated in Figure 2B by our novel software program VennPlex, demonstrating the number of genes up-regulated, down-regulated and contra-regulated in each Venn diagram sector. The Venn diagram drawn by VennPlex is able to provide the end-user with considerably more detailed and immediately-visual information than the diagram generated by Venny. In Figure 2A, a simple Venn format structure demonstrates that 532 transcripts were significantly altered in astrocytes exposed to 1% O_2_ ambient tension compared to 20% O_2_ tension. However, the Venn diagram drawn by VennPlex can demonstrate instantly that, among these 532 significantly-regulated transcripts, 190 were up-regulated while 342 were down-regulated compared to 20% O_2_ tension (Figure 2B). Manual sorting of such differentially-regulated transcripts [20] is often time consuming for the general end-user that does not have access to expensive and sophisticated software suites. In addition, there were a total of 60 transcripts significantly regulated at all oxygen tensions, as shown in the intersection between all sets in Figure 2A. With VennPlex it is facile to discover that among these 60 transcripts, 27 transcripts were commonly up-regulated (Table S1), 23 were commonly down-regulated (Table S3), and 10 were multiply and diversely regulated by the different ambient O_2_ tensions (Table S2: Figure 2B). With respect to these commonly or diversely regulated transcripts, the details including the gene symbols and corresponding z-ratios in each group can be easily accessed by saving the data in regions 18, 19 and 20, respectively (Figure 1B: Tables S1, S2, S3). Indicative of the ability of VennPlex to rapidly assist in a more comprehensive appreciation of physiological relevance, it is interesting to note that within the many transcripts responsive to multiple oxygen tensions, several have been recently demonstrated to be controlled by oxygen levels in the central nervous system. For example, the expression of chemokine orphan receptor 1(*Cmkor 1*) [22], DNA-damage-inducible transcript 3 (*Ddit 3*) [23] and prostacyclin synthase (*Ptgis*) [24] were found to be enhanced after cerebral ischemia. These genes were also found to be up-regulated in astrocytes exposed to all oxygen tension groups with different change ratios (Table S1). To compare multiple simultaneous experiments with genomic data is often time consuming for biological scientists; therefore we have shown that using a robust platform application such as VennPlex, subtle nuances in expression data can be easily identified and extracted.

## Discussion

With the ever-increasing prevalence of larger and more complex datasets, rapidly generated by “*omics*” studies, advanced bioinformatics tools are needed to evolve alongside “*omics*” data [25]. VennPlex is a user-friendly program for easily visualizing and categorizing multiple and highly complex biological data sets, making the analysis of multiple parallel datasets produced by microarray, Genome Wide Association Studies (GWAS), proteomics, or metabolomics, easy to distinguish and specifically filter. This application can be downloaded and used on any computer using a MicroSoft™ Windows™ operating system. This program is freely available to the scientific community on the National Institute on Aging website (www.irp.nia.nih.gov/bioinformatics/vennplex.html). VennPlex has various features, such as simple Excel™-based data input/output, and representation of the regulation pattern within the datasets. Depending on end-user feedback, there are several potential advancements that we could expand upon in future VennPlex iterations, such as expanding the parallel set analysis mode above four to potentially 6–10 parallel analytical sets, and a potential version of VennPlex as an Excel plugin to facilitate data import/export.

One potential application of the VennPlex program was shown in this study. VennPlex helped us to easily visualize and categorize the differentially expressed gene sets in astrocytes that were sensitive to multiple levels of ambient oxygen tension. The Venn diagram generated by VennPlex assisted a direct overview of how astrocytes respond to multiple ambient oxygen tensions. More transcripts were shown to be altered when astrocytes were exposed to 1% O_2_ tension, representing the most severe hypoxia condition, compared to 4, or 9% ambient O_2_. The Venn diagram also demonstrated that there were more genes down-regulated in the 1% O_2_ tension group, suggesting that the astrocytes’ activities may be attenuated when responding to a very low oxygen level. In addition, it is interesting to note that many multi-tension-responsive transcripts, especially those genes up-regulated in all oxygen tension groups, have been strongly implicated in cytoprotective roles during CNS ischemic events [22], [24]; therefore VennPlex allows the rapid identification and functional classification of transcripts potentially responsible for neurological ageing/damage repair processes. The capability to generate such an in-depth appreciation of the ambient oxygen related gene transcription alterations in astrocytes may therefore assist in the rational design of pharmacotherapeutics that target oxidative stress-related neurodegenerative disorders. While other Venn diagram-generating programs exist that can employ multiple input sets and generate proportional areas, we feel that our novel VennPlex software will be an especially valuable tool for the research community as it can be used for any research purpose that requires visualization and interpretation of the numerical commonalities and differences between large datasets with variety of numerical regulatory patterns.

In summary, VennPlex has many practical features that can facilitate scientific research, such as comparison and visualization of the changing numerical patterns for shared experimental factors within the datasets, ease of data input (*i.e.* the ability to input raw excel data), and versatile output features, especially exporting the numerical data variations within each region in the overall Venn diagram.

## Supporting Information

Table S1 **Significantly up-regulated transcripts common between 1, 4, 9% O_2_ tension versus 20% O_2_.** Official gene symbols are employed to demonstrate the significantly up-regulated genes populating the Venn diagram region 18, depicted in Figure 2B.
(DOC)

Table S2 **Significantly contra-regulated transcripts in 1, 4, 9% O_2_ tension versus 20% O_2_.** Official gene symbols are employed to demonstrate the significantly contra-regulated genes populating the Venn diagram region 19, depicted in Figure 2B.
(DOC)

Table S3 **Significantly down-regulated transcripts common between 1, 4, 9% O_2_ tension versus 20% O_2_.** Official gene symbols are employed to demonstrate the significantly down-regulated genes populating the Venn diagram region 20, depicted in Figure 2B.
(DOC)
