# Supplementary material for: VennPlex–A Novel Venn Diagram Program for Comparing and Visualizing Datasets with Differentially Regulated Datapoints
Source: PLoS One. 2013 Jan 7;8(1):e53388. doi: 10.1371/journal.pone.0053388 (PMC3538763; doi:10.1371/journal.pone.0053388)
Supplement: Table S1 — Significantly up-regulated transcripts common between 1, 4, 9% O2 tension versus 20% O2. Official gene symbols are employed to demonstrate the significantly up-regulated genes populating the Venn diagram region 18, depicted in Figure 2B. (DOC) [file pone.0053388.s001.doc]

**Table S1. Significantly up-regulated transcripts common between 1, 4, 9% O_2_ tension versus 20% O_2_.** Official gene symbols are employed to demonstrate the significantly up-regulated genes populating the Venn diagram region 18, depicted in Figure 2B.

| Gene symbol | 1% O_2_ tension z ratio | 4% O_2_ tension z ratio | 9% O_2_ tension z ratio |
| --- | --- | --- | --- |
| Acta1 | 8.58 | 1.87 | 3.52 |
| LOC500965 | 4.75 | 2.36 | 3.35 |
| Pgk1 | 4.67 | 1.66 | 3.18 |
| Aldoa | 4.35 | 1.6 | 2.01 |
| Atf4 | 4.23 | 2.38 | 1.77 |
| LOC307731 | 4.23 | 1.76 | 3.29 |
| Ddit3 | 4.21 | 2.31 | 1.87 |
| Ldha | 4.16 | 2.05 | 2.65 |
| LOC500271 | 4.05 | 2.13 | 2.63 |
| LOC498099 | 4.05 | 2.59 | 2.73 |
| LOC295452 | 3.67 | 2.15 | 2.25 |
| LOC290634 | 3.65 | 1.9 | 2.49 |
| LOC499433 | 3.53 | 1.92 | 2.72 |
| LOC365954 | 3.39 | 2.25 | 3.25 |
| LOC295423 | 3.38 | 2.29 | 3.37 |
| LOC364848 | 3.38 | 2.35 | 3.16 |
| LOC498618 | 3.27 | 1.65 | 1.62 |
| LOC498881 | 3.21 | 1.8 | 2.83 |
| LOC498019 | 3.12 | 2.13 | 2.45 |
| LOC500104 | 3.08 | 2.17 | 2.7 |
| Lamr1 | 2.65 | 2.1 | 1.92 |
| LOC502063 | 2.6 | 1.76 | 1.88 |
| LOC299622 | 2.39 | 1.85 | 1.92 |
| Ubb | 2.24 | 1.56 | 2.23 |
| Ptgis | 1.96 | 3.37 | 4.14 |
| Cmkor1 | 1.85 | 1.7 | 3.49 |
| LOC294700 | 1.78 | 1.61 | 2.25 |
