# Supplementary material for: VennPlex–A Novel Venn Diagram Program for Comparing and Visualizing Datasets with Differentially Regulated Datapoints
Source: PLoS One. 2013 Jan 7;8(1):e53388. doi: 10.1371/journal.pone.0053388 (PMC3538763; doi:10.1371/journal.pone.0053388)
Supplement: Table S2 — Significantly contra-regulated transcripts in 1, 4, 9% O2 tension versus 20% O2. Official gene symbols are employed to demonstrate the significantly contra-regulated genes populating the Venn diagram region 19, depicted in Figure 2B. (DOC) [file pone.0053388.s002.doc]

**Table S2. Significantly contra-regulated transcripts in 1, 4, 9% O_2_ tension versus 20% O_2_.** Official gene symbols are employed to demonstrate the significantly contra-regulated genes populating the Venn diagram region 19, depicted in Figure 2B.

| Gene symbol | 1% O_2_ tension z ratio | 4% O_2_ tension z ratio | 9% O_2_ tension z ratio |
| --- | --- | --- | --- |
| Adm | 2.84 | -2.57 | -2.58 |
| Mdm2 | 1.53 | -1.59 | -1.75 |
| Nedd9 | -1.63 | 2.2 | 2.05 |
| Ppp1r14b | -1.7 | -2.01 | 1.65 |
| Gstp1 | -1.71 | 4.05 | -3.35 |
| Mtch2 | -1.81 | 1.7 | 1.64 |
| Ndr4 | -2.02 | 2.04 | -1.69 |
| Tnfrsf11b | -2.29 | 5.37 | 2.36 |
| LOC500987 | -3.69 | -1.61 | 2.15 |
| Dspg3 | -4.14 | 4.12 | 2.62 |
