# Supplementary material for: VennPlex–A Novel Venn Diagram Program for Comparing and Visualizing Datasets with Differentially Regulated Datapoints
Source: PLoS One. 2013 Jan 7;8(1):e53388. doi: 10.1371/journal.pone.0053388 (PMC3538763; doi:10.1371/journal.pone.0053388)
Supplement: Table S3 — Significantly down-regulated transcripts common between 1, 4, 9% O2 tension versus 20% O2. Official gene symbols are employed to demonstrate the significantly down-regulated genes populating the Venn diagram region 20, depicted in Figure 2B. (DOC) [file pone.0053388.s003.doc]

**Table S3. Significantly down-regulated transcripts common between 1, 4, 9% O_2_ tension versus 20% O_2_.** Official gene symbols are employed to demonstrate the significantly down-regulated genes populating the Venn diagram region 20, depicted in Figure 2B.

| Gene symbol | 1% O_2_ tension z ratio | 4% O_2_ tension z ratio | 9% O_2_ tension z ratio |
| --- | --- | --- | --- |
| Slc27a1 | -1.52 | -2.14 | -1.86 |
| Cd38 | -1.59 | -1.54 | -2.66 |
| Nid2 | -1.63 | -1.87 | -1.99 |
| Prelp | -1.63 | -3.42 | -2.16 |
| Plekhb2 | -1.65 | -1.51 | -1.73 |
| Csad | -1.68 | -3.05 | -1.61 |
| Gnpat | -1.75 | -2.15 | -1.57 |
| Tec | -1.88 | -2.56 | -1.58 |
| LOC313974 | -1.9 | -1.58 | -2.19 |
| Giot1 | -1.99 | -3.42 | -1.57 |
| Lgi4 | -2.04 | -1.71 | -3.2 |
| Sec24d | -2.07 | -1.63 | -2.87 |
| LOC499196 | -2.08 | -1.69 | -3.12 |
| Gpr37l1 | -2.1 | -2.85 | -2.3 |
| Olig1 | -2.1 | -3.92 | -1.82 |
| LOC317575 | -2.12 | -2.99 | -2.53 |
| LOC315804 | -2.15 | -1.86 | -1.73 |
| MGC72974 | -2.17 | -1.5 | -1.63 |
| Serpinb1a | -2.23 | -2.26 | -1.52 |
| Nr2f1 | -2.24 | -1.53 | -1.66 |
| Nrp1 | -2.31 | -2.46 | -2.01 |
| LOC499856 | -2.32 | -2.85 | -1.59 |
| Tnmd | -2.38 | -1.7 | -3.78 |
| Podxl | -2.75 | -1.52 | -1.93 |
| LOC498356 | -2.82 | -3.22 | -2.1 |
| Ttyh1 | -2.95 | -1.74 | -2.03 |
| Cyp26b1 | -3 | -2.69 | -3.89 |
| Tcfap2b | -3.02 | -2.64 | -3.31 |
| MGC94018 | -3.21 | -2.15 | -2.43 |
| LOC294789 | -3.57 | -4.41 | -3.02 |
| Cldn11 | -4.11 | -3.56 | -2.04 |
| Atp1a2 | -4.53 | -4.23 | -2.79 |
| Enpp2 | -4.53 | -5.52 | -3.83 |
